# Solvents and sustainable chemistry

**DOI:** 10.1098/rspa.2015.0502

**Published:** 2015-11-08

**Authors:** Tom Welton

**Affiliations:** Department of Chemistry, Imperial College London, London SW7 2AZ, UK

**Keywords:** solvent, sustainability, green chemistry, sustainable chemistry, process chemistry

## Abstract

Solvents are widely recognized to be of great environmental concern. The reduction of their use is one of the most important aims of green chemistry. In addition to this, the appropriate selection of solvent for a process can greatly improve the sustainability of a chemical production process. There has also been extensive research into the application of so-called green solvents, such as ionic liquids and supercritical fluids. However, most examples of solvent technologies that give improved sustainability come from the application of well-established solvents. It is also apparent that the successful implementation of environmentally sustainable processes must be accompanied by improvements in commercial performance.

## 1. Introduction

In 1987, the United Nations defined sustainable development as development that enabled the current generation to meet its own needs, without compromising the ability of future generations to meet their needs [1]. *Sustainable Chemistry is the implementation of the concept of sustainability in the production and use of chemicals and chemical products* and *the application of chemistry and chemical products to enable sustainable development*. The first part of this overlaps significantly with green chemistry—the reduction or elimination of the use or generation of hazardous substances in the design, manufacture and application of chemical products [2,3,4]. The second part makes it clear that *the benefits of modern chemistry and chemical products should be made available to all communities*. Horváth and co-workers have described sustainable chemistry as: *resources including energy should be used at a rate at which they can be replaced naturally* and *the generation of wastes cannot be faster than the rate of their remediation* [5]. However, it is only by commercial production that chemical products impact upon people's lives or the environment. If the product is too expensive, it will not be bought by users; if the transaction is not profitable, it will cease to be supplied. In either case, the product will fall out of use and will not be sustainable. Hence, we should add to Horváth's description that: *a sustainable chemical product* *should be supplied at a price that enables it to be accessed by its users while at the same time being commercially viable for its producers*. Finally, there is some confusion about whether sustainability should be considered to be an absolute or relative term. This arises because while it is possible for a product or process to be absolutely unsustainable, it is not possible to be absolutely sustainable. This is because the external environment and economy change and as new conditions come about something that was once considered sustainable may no longer be so, or through innovation for it to be superseded by a more sustainable alternative.

Government regulation has played a significant role in the protection of the environment. Emission controls have been used for over 150 years (http://www.legislation.gov.uk/ukpga/Vict/10-11/34), and the use of specific classes of compounds has been eliminated, such as under the Montreal Protocol on Substances that Deplete the Ozone Layer (http://ozone.unep.org/en/treaties-and-decisions/montreal-protocol-substances-deplete-ozone-layer). Regulatory controls are probably to continue and increase, as with the European Union regulation for Registration, Evaluation, Authorization and restriction of Chemicals (REACH) (http://echa.europa.eu/web/guest/regulations/reach). However, by seeking chemicals and chemical production methods that are both environmentally and commercially sustainable, sustainable chemistry goes beyond that which can be achieved through regulation alone.

Solvents have many uses, both commercial and domestic. In the chemicals industry, solvents are used in the production of chemicals as media for chemical reactions and for chemicals separation/purification. Here, I attempt to demonstrate how appropriate selection of solvents for chemicals processing has been used to improve the sustainability of these processes using examples that have been, to the best of my knowledge using publicly available information, in commercial use at some time. These have been selected for illustrative purposes and are not an exhaustive collection of all the available examples in the literature.

## 2. Green metrics

The sustainability of a chemical product or process is necessarily the result of a complex interaction of environmental, technological and economic factors and is difficult to predict. Guides are required to provide means to select probably useful avenues for further research and development. Early stage techno-economic modelling techniques are relatively well established [6]. Measures of environmental sustainability are less well developed.

Life cycle assessment (LCA) is considered the gold-standard environmental impact assessment for any product or process. LCA is a collection of techniques designed to assess the environmental impacts associated with all stages of a product's creation, use and disposal, including any reuse or recycling, from ‘cradle to grave’ [7–9]. While LCA attempts to be comprehensive, it is sensitive to the amount and quality of data available and to choices made about precisely what is included, and how, in the analysis. Consequently, different analyses of the same product or process can come to different conclusions. LCA can also be prohibitively expensive. LCA approaches can be relevant to products and processes either already in commercial application or those at high technology readiness levels. However, LCA is not a useful tool for those engaged earlier in the innovation pipeline. For these, simpler metrics are required [10].

The simplest green metric is *Atom Economy* [11,12]. This was introduced to focus chemists' attention away from yield as the only measure of reaction efficiency and on to the inherent efficiencies of different types of reactions. It measures the ratio of the mass of the final product to the sum of the masses of all the starting materials, expressed as a percentage. Simple addition and isomerization reactions in which all the starting materials become part of the product have 100% atom economy, whereas substitutions and eliminations always have lower atom economies. The advantage of atom economy is that it is a simple concept that can always be calculated if the reaction stoichiometry is known. However, its usefulness is limited because it only considers the stoichiometry of the reaction and does not take into account the yield of the desired product. *Reaction Mass Efficiency* (the ratio of the mass of the isolated product to the total mass of all the reactants, expressed as a percentage) was introduced in order to take yield into account [13]. However, neither of these metrics accounts for the fates of ancillary chemicals used in the reaction, such as solvents.

A group of simple mass-based metrics have been developed to measure the ‘greenness’ of a chemical process. The first of these was the *Environmental Factor* (E-factor), introduced by Roger Sheldon [14,15]. The E-factor is the ratio of the amount of waste generated by the process compared with the amount of product obtained (mass of waste/mass of product) with lower values preferable. Waste is defined as everything produced from the process that is not the desired product, including ancillary materials such as solvents. Its simplicity leads to it being the most frequently used of all green metrics. It does not differentiate waste by its potential to cause harm in the environment, so a process that gives a large amount of water or NaCl as a by-product will score worse than one that produces a small amount of a highly toxic and environmentally persistent by-product. This led to the introduction of *Effective Mass Yield* (EMY; the percentage of the mass of product relative to the mass of all non-benign materials used in its synthesis) [16], which does not include environmentally benign compounds in the calculation of the amount of waste.

In 2001, the ACS Green Chemistry Institute Pharmaceutical Round Table (ACS GCI-PR) (http://www.acs.org/content/acs/en/greenchemistry/industry-business/pharmaceutical.html) advocated *Process Mass Intensity* (PMI; the ratio of the total mass in a process or process step to the mass of the product) as a measure of the greenness of a process. Its commitment to PMI as the best of the simple metrics for driving behaviours towards the development of more sustainable processes was reaffirmed a decade later [17]. This preference was justified on the basis that mass-based metrics are generally preferable and that, of these, PMI takes into account the yield of the product achieved, all the materials used in the synthesis, including all ancillary materials and those used in the product isolation and purification, which can be far greater than those used in the reaction itself. Although simply mathematically related to the E-factor, the ACS GCIPR believes that PMI is preferable, because it focuses attention upon optimization of resource use (inputs) rather than the waste generated by a process (outputs), which is the emphasis of the E-factor. It proposes that this is particularly important for discussions regarding the economics of chemicals production: ‘Focusing on reducing waste helps companies to reduce costs, but focusing on efficiency also enables innovation to create additional value’ [17]. It also provides evidence that PMI is a better high-level proxy for LCA than other commonly applied metrics, particularly when applied across value chains. PMI has also been endorsed and its use encouraged in a recent editorial in *Organic Process Research & Development* [18].

There have been attempts to bring collections of measures together, e.g. *Environment, Health and Safety* (EHS) [19] or *Ecological and Economic Optimization Methods* [20]. EHS assigns a score for a process or product based upon environmental (persistency, air hazard, water hazard), health (acute toxicity, chronic toxicity, irritation) and safety (release potential, fire or explosion risk, reaction or decomposition potential) considerations, with low scores preferred. These multi-parameter approaches offer greater sophistication, but they are necessarily more complex to apply.

When there are many different metrics that can be applied to analyse the greenness of a product or process, the obvious question is which is best [21]. Each metric has its own strengths and there is no general consensus on which of these is best. It has been noted that it is better to think of which metric is more appropriate to any given situation rather than thinking that one metric will always be better than another [22] or that a toolkit approach is preferred [23]. Over the last few years, I have taught a course at Imperial College London during which the students analyse a literature claim of improved greenness. Over the years and several hundred papers analysed, it is rare for such claims to be accompanied by quantitative green analysis, nor is enough information included to allow the reader to calculate these values independently. So first it should be noted that any quantitative analysis is better than none at all. However, these students have found that it is best to use several of the available metrics together. Their analyses show that, when a process scores well for one metric, but poorly for another, this can be used to understand the process more fully and to identify points for improvement.

## 3. ‘Green’ solvents

Many commonly used solvents have been recognized as being of environmental concern. These concerns arise in three areas: the source and synthesis of the solvent itself; its properties in use, including accidental discharge; and finally disposal. A great deal of the literature of solvent use advocates that one solvent or class of solvents should be regarded as inherently ‘green’. Solvents and solvent classes that have been suggested as ‘green’ solvents include water [24–32], supercritical fluids [33–40], gas expanded liquids [41], ionic liquids [42–49], liquid polymers [50–56] and solvents derived from biomass [57–66]. This is based on the idea that replacing a ‘non-green’ solvent in a process with a ‘green’ solvent necessarily improves its environmental performance. This, in turn, has led to debates in the literature about which of these solvents is greener [67]. Ionic liquids have, with their often complex syntheses and toxicities, been particularly criticized in this respect [67,68], although so has water [69].

The selection of the solvent for a reaction can dramatically affect the reaction outcome [70]. Hence, it is possible that a replacement of a ‘non-green’ solvent by a ‘green’ solvent could lead, for example, to a lower yield of the product and greater waste, or the need for harsher operating conditions that require more energy. In these cases, the process could become less environmentally sustainable overall. In order to thoroughly understand how a solvent change can affect the sustainability of a process, it is necessary to consider all its impacts on the overall process. Hence, the idea that a liquid can be regarded as inherently ‘green’ is somewhat naive, even irrelevant. What matters is whether the use of one solvent or solvent system rather than another can give a more sustainable process and/or product (see below).

Notwithstanding the above, it is possible to make some points about the general acceptability of different solvents. A number of solvent selection guides have emerged from the pharmaceutical industry, i.e. ACS GCI-PR (http://www.acs.org/content/dam/acsorg/greenchemistry/industriainnovation/roundtable/acs-gci-pr-solvent-selection-guide.pdf), GSK [71–73], Pfizer [74] and Sanofi [75]. While different in detail, these all share the aim of distilling a great deal of information into an easily used form. There is good general agreement between the guides, but they do not all come to precisely the same conclusions as to how desirable every solvent might be. This is not a problem if these are treated as general guides that can be applied quickly and easily and not as definitive statements as to the applicability of any particular solvent in any particular process.

The first of these guides came from SmithKline Beecham [71]. Earlier solvent selection tools were directed at solvents as cleaning agents and did not consider issues of importance in pharmaceutical production, such as process safety. Their initial guide was based upon: impacts on incineration—heat of combustion, emissions on incineration, water solubility; ease of recycle—boiling point, number of solvents with similar boiling points, formation of azeotropes; ease of drying—reactivity, water solubility; ease of biotreatment—fate in wastewater treatment; volatile organic compound potential—vapour pressure, boiling point; aqueous environmental impact—acute toxicity, log octanol/water partition coefficient; atmospheric environmental impact—rate of photolysis, photochemical ozone creation potential (POCP), odour threshold; health impact, acute or chronic; workplace exposure potential; and process safety—flash point, conductivity, risk of peroxide formation. Thirty-five solvents were ranked according to these criteria and colour coded in respect of environmental waste, environmental impact, health and safety. Later versions of the guide, published by GSK, added LCA [71,72] and regulatory concerns [71,73].

The Pfizer ‘traffic light’ solvent selection guide has three categories (preferred, usable and undesirable) of solvent [74]. Its methodology considered: worker safety—carcinogenicity, mutagenicity, reprotoxicity, skin absorption/sensitization, toxicity; process safety—flammability, vapour pressure, static charge, peroxide formation, odour; environmental and regulatory concerns—ecotoxicity, ground water contamination, EHS restrictions, ozone depletion potential, photoreactive potential. Its methodology followed from the work of Fischer and co-workers [76], who applied the EHS method to a number of solvents. A website has been built [77], which allows one to apply this methodology to solvents not originally included (e.g. when low molecular weight siloxanes [78] were proposed as replacements for non-polar solvents). The Pfizer selection guide does not try to give absolute measures, but makes relative judgements. So while ethyl acetate or 2-methyltetrahydrofuran are proposed as possible replacements for dichloromethane, dichloromethane is proposed as a possible replacement for even less desirable chlorinated solvents, such as chloroform. When *Organic Process Research & Development* took the stance that ‘green chemistry is good process chemistry’ it recommended solvent replacements for ‘strongly undesirable solvents’ from the Pfizer solvent selection guide [79].

The Sanofi guide compares solvents in different chemical classes (alcohols, ketones, esters, ethers, hydrocarbons, halogenated, polar aprotic, bifunctional and miscellaneous) and gives these a ranking of banned, substitution requested, substitution advisable and recommended [75]. The overall ranking was derived from consideration of safety, occupational health, environment, quality and industrial constraints, the results of which were also separately reported. Sanofi found that recommending preferred solvents within a family is relatively straightforward, so attempted to recommend at least one solvent from each family.

The Innovative Medicines Initiative (IMI)-Chem21, a public–private partnership of pharmaceutical companies, universities and small-to-medium enterprises supporting research into sustainable pharmaceuticals manufacturing (http://www.chem21.eu/), compared these solvent selection guides [80]. The authors transformed the guides into a form in which direct comparisons could be made and brought these together into a single guide. This is a six-point scale of recommended, recommended or problematic, problematic, problematic or hazardous, hazardous and highly hazardous solvents (table 1).

**Table 1.** Combined green solvent selection guide ranking [80].

recommended	recommended or problematic	problematic	problematic or hazardous	hazardous	highly hazardous
water ethanol 2-propanol 1-butanol ethyl acetate 2-propyl acetate 1,1-dimethylethyl acetate anisole sulfolane	methanol *tert*-butyl alcohol benzyl alcohol ethylene glycol acetone butanone 4-methyl-2- pentanone cyclohexanone methyl acetate acetic acid acetic anhydride	2-methyltetrahydrofuran heptane methylcyclohexane toluene xylenes chlorobenzene acetonitrile 1,3-dimethyltetrahydropyrimidin- 2(1*H*)-one dimethyl sulfoxide	2-methoxy-2- methylpropane tetrahydrofuran cyclohexane dichloromethane formic acid pyridine	diisopropylether 1,4-dioxane dimethyl ether pentane hexane dimethylformamide *N*,*N*-dimethylacetamide 1-methyl-2-pyrrolidone methoxy ethanol triethanolamine	diethylether benzene chloroform carbon tetrachloride dichloroethane nitromethane

These green solvent guides do not consider the use to which the solvent will be put, yet the ability of the selected solvent to be effective for this use is of primary importance. One way of dealing with this is to combine the environmental assessment with estimates of the ability of the solvent to promote a reaction. There is a long history of the study of solvent effects on chemical reactivity [70]. Attempts have been made to generate software tools that combine consideration of properties related to this with green selection criteria [81]. However, these two sets of criteria are mostly treated separately. Another way that has been used to take into account the role that the solvent plays is to restrict the guide to a particular application or to target the elimination of a particular solvent, such as CH_2_Cl_2_ in chromatography [82,83], amide coupling [84], reductive amination [85] and olefin metathesis reactions [86].

## 4. Sustainable solvent use

As green chemistry spread some tension between those working in the field, largely in academia, and those working in process chemistry, largely in industry, began to emerge [87,88]. The target of creating low-waste, efficient chemistry that delivers products in an economically viable way is not new and both endeavours are equally capable of contributing to sustainable chemical solutions. Indeed, the sustainability of any chemical synthesis process equally depends upon finding chemical engineering solutions [89–92].

Reports of direct replacement in industry of a solvent by an alternative in an existing commercial process just for the purpose of creating a greener process are rare in the literature. In pharmaceuticals production, the need for renewed regulatory approval of the product, particularly in multiple jurisdictions, after a significant change in the synthesis process can create a barrier to such replacements [93]. In bulk chemicals production, the cost of replacing large-scale production plant equipment can generate a commercial barrier to such replacements. Consequently, any changes must be accompanied by economic improvements in the process to be able to compensate for these expenses.

The principal reason why solvents are of great environmental concern is that they are used in vast quantities. It is normal in chemicals production for the solvent to be used in large excess in comparison with the reactants and products. It has been estimated that at least half of the material used in the production of a pharmaceutical is solvents [17]. Hence, it has been asserted that the ideal green reaction has no solvent at all [94–96]. Many bulk chemicals processes are conducted in the gas phase without solvents [97]. While the concept is not new [98], the attempt to eliminate solvents entirely has led to research into all-solid reactions initiated by grinding, ‘mechanochemistry’ [99,100]. Reactions involving the breaking and making of covalent bonds have been studied academically, but much of the recent literature has concentrated on changes in intermolecular forces and interconversions between polymorphs [101,102]. However, the use of all-solid reactions has not yet found widespread industrial application. Also, for many syntheses, most solvent is used for product isolation and purification and eliminating added solvents for the reaction itself only has a minor effect on the amount of solvent used.

Whenever a homogeneous liquid mixture is present, there is a solvent. The solvent is the major component of the liquid mixture (solution) and it is usually a liquid under the conditions described when pure. The minor components of the solution are the solutes. The solvent can have more than one function, such as being one of the starting materials for a reaction. It is common for these conditions to be described as ‘solvent-free’, but this is incorrect. However, in such cases it is unnecessary to add another liquid to act as the solvent, which is what the ‘solvent-free’ label is often used to indicate. This can improve the environmental performance of a reaction, but does not necessarily do so.

The E-factor has been applied to different industrial sectors and it was shown that the proportion of waste generated by pharmaceuticals production was much greater than fine or bulk chemicals production [14]. GSK has estimated that more than 70% of the waste associated with pharmaceutical production is solvents [103]. This can be attributed to the greater number of steps in the synthesis of a complex pharmaceutical. As also shown by PMI, it is not the number of transformations in a complex synthesis but the number of isolations of intermediate products that leads to large amounts of solvent waste [13,17]. This has led to an interest in ‘one-pot’, ‘multi-component’, ‘cascade’ ‘tandem’, ‘convergent’, ‘telescoped’ and similar synthetic approaches.

Solvent recovery, usually by distillation, forms part of many strategies for the reduction of solvent wastes. However, it is not always preferable to incineration with energy recovery. It has been found that the preferred option largely depends upon the original production of the solvents. If the production of the solvent has a low environmental impact then incineration may be the best option; if it has a high environmental impact then solvent recovery is usually the best option [104]. New technologies, such as solvent-resistant nanofiltration, can provide lower energy and more cost effective separations and tip the balance in favour of solvent recovery.

### (a) The development of Pfizer's production process for sildenafil citrate, Viagra [105,106]

The reduction of solvent use is a normal aim of the development of the commercial synthesis of a pharmaceutical. For sildenafil citrate a reduction in solvent use from 1300 l kg^−1^ for the original medicinal chemistry route to 7 l kg^−1^ for the final commercial route (with solvent recovery and recycling) was achieved. As well as using less solvent, the commercial process also uses less harmful solvents.

The Viagra story illustrates some important points [105]. The purpose of the initial medicinal chemistry route is to generate just enough product for screening to identify a compound of potential interest. This synthesis will be conducted alongside many others for compounds with similar structures, the majority of which will not be taken forward for further development. Until a pharmaceutical lead has been identified, there is no driver to further develop the synthesis. Indeed, efforts to optimize reactions to improve their environmental efficiency at this stage could lead to overproduction of compounds that will not be taken forward and poorer resource efficiency and greater waste overall. What is crucial at this stage, and indeed in other research laboratories such as those in universities, is that the highest quality of chemical inventory control and waste management are used to minimize the environmental impacts of the laboratory's activities.

**Scheme 1.**
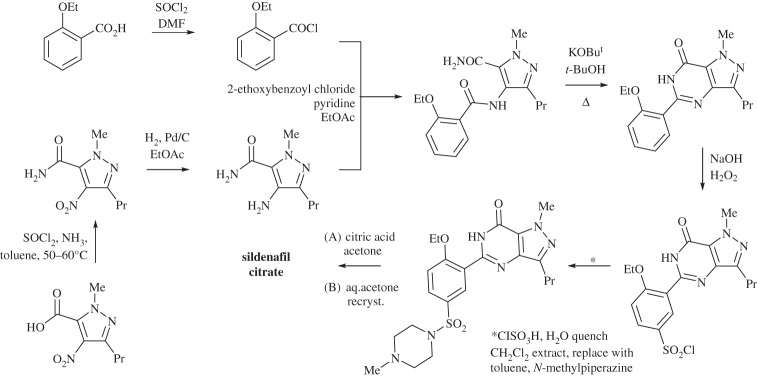
The optimized medicinal chemistry route to sildenafil citrate (Viagra) [105].

Once the sildenafil citrate had been identified as a potential pharmaceutical lead, the next step was to find a safe and effective route to generate kilograms of the compound, which was the first time that the environmental consequences of choice of synthetic protocols were considered. The attrition rate for pharmaceutical leads during the preclinical and phase 1, 2 and 3 trials and registration is so great that less than 5% are approved for use [107]. Hence, at this stage no dramatic changes to the synthesis were made and the foci of this were the reduction in the use of the most toxic reagents and to find reactions to give more efficient steps. This optimized medicinal chemistry route (scheme 1) replaced a tin chloride-based reduction with a catalytic hydrogenation, used thionyl chloride in stoichiometric quantities in toluene rather than as a solvent and gave a large reduction in the use of dichloromethane [105].

**Scheme 2.**
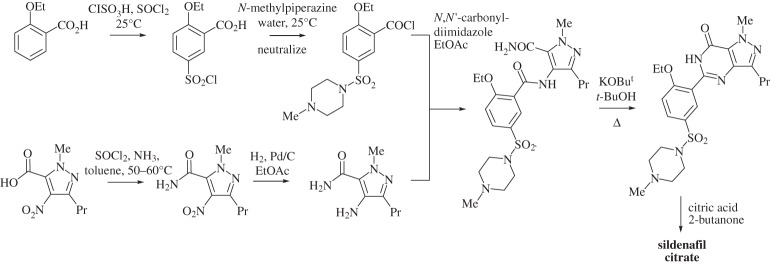
The commercial route to sildenafil citrate (Viagra) [105].

Once sildenafil citrate had been confirmed as the commercial pharmaceutical, a new production synthesis was developed. The replacement of the almost entirely linear process with a convergent synthesis led to a more efficient overall process, while moving reactions involving toxic materials to the beginning of the process and cleaner steps to the end reduced the need for multiple purifications of the final product and gave an attendant reduction in solvent use (scheme 2) [105].

Although the atom economy of the commercial route was slightly worse than that of the medicinal chemistry route, 54% rather than 56%, the RME was increased from 10% to 26% [105]. However, neither of these metrics takes into account the dramatic reduction in solvent use. There are some differences in the information presented in the two papers, but taking the more detailed data provided in the earlier paper [105], the PMI of the optimized medicinal chemistry route is of the order of 134, which was reduced to 16 for the commercial route. The majority of this change was due to the reduction in solvent use (from 124 kg kg^−1^ product to 12 kg kg^−1^ product), which dwarfs the reduction in use of reagents (10 kg kg^−1^ product to 4 kg kg^−1^ product). Finally, solvent waste was further reduced by solvent recovery and recycling [105]. The number and types of solvents was also changed to ones of lower environmental concern [105].

## 5. Biocatalysts in water

Biocatalysis has become a standard synthetic technique across a wide range of the chemicals and pharmaceutical industries [108,109]. While enzyme catalysis in non-aqueous solvents has been known for a long time [110], water is the solvent of choice for biocatalytic processes. Hence, the use of enzyme-catalysed reactions is often accompanied by a replacement of non-aqueous solvents with water and so is included here. The use of enzymes in water has also enabled improvements in other environmental impacts of many processes [111–113].

### (a) Pfizer's chemoenzymatic synthesis of pregabalin

**Scheme 3.**
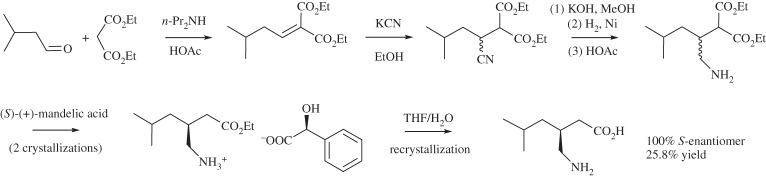
The original commercial synthesis of pregabalin [114].

Pregabalin, (*S*)-3-(aminomethyl)-5-methylhexanoic acid, is a treatment for central nervous system disorders. Its original commercial synthesis (scheme 3) began with a Knoevenagel condensation, followed by cyanation, introducing a chiral centre as a racemic mixture, then hydrolysis, decarboxylation and hydrogenation in methanol to yield a γ-aminoacid [114]. (*S*)-(+)-Mandelic acid was then added in aqueous *iso*-propyl alcohol (*i*-PrOH) to give a classic chiral resolution and the resulting diastereomeric salt was split by recrystallization from aqueous THF, followed by recrystallization from *i*-PrOH to yield pure pregabalin. This malonate route was compared all the way to pilot plant scale with another that used γ-isobutylglutaric acid. Costs, throughput and the amount of waste generated were largely comparable, but the γ-isobutylglutaric acid route used chloroform and so was rejected because the necessary control measures would have led to greater capital outlay. This demonstrates how the avoidance of hazardous solvents can reduce the cost of chemicals production.

**Scheme 4.**
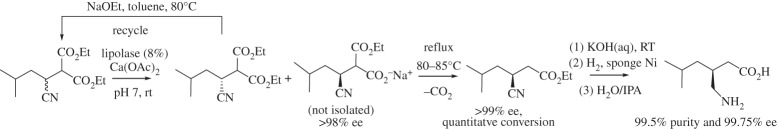
The enzymatic synthesis of pregabalin [116].

The generation of the γ-aminoacid as a racemic mixture and the need to obtain the enantiomerically pure pregabalin led to both waste of the compound itself and the use of large amounts of solvents. Reports can be found in the literature from both Pfizer [115] and Dowpharma [116] of the development of asymmetric hydrogenation-based routes to avoid this problem. However, Pfizer's eventual solution was an enzyme-catalysed process (scheme 4) [117]. An enzyme-catalysed kinetic resolution hydrolyses one of the esters of the β-cyano diester to yield the sodium salt of the carboxylic acid. The unreacted diester is then recycled and racemized in toluene to be reused, while the carboxylic acid is thermally decarboxylated in the aqueous solution. This yields the β-cyano ester as a water-insoluble oil, which separates leaving the majority of the impurities in the aqueous layer. Hydrogenation in aqueous *i*-PrOH completes the synthesis. The authors report that this led to a reduction of the E-factor from 86 for the original commercial route to 17 for the new route and a reduction in solvent use from 50 kg kg^−1^ product to 6.2 kg kg^−1^. Perhaps some concern remains at the use of toluene in the racemization process, but the environmental performance of the synthesis has been significantly improved.

### (b) Mitsubishi Rayon's synthesis of acrylamide

Acrylamide is a commodity chemical used as the monomer for the polymer polyacrylamide. It is prepared by the hydration of acrylonitrile. The traditional synthesis used copper catalysts and exhibited problems such as incomplete reaction of the acrylonitrile, requiring its recovery from the product mixture, and the formation of by-products, such as acrylic acid nitrylotrispropionamide, ethylene cyanohydrin and polymers of both the starting material and product [118]. The biotransformation using nitrile hydratase enzymes with the addition of iron(II) sulfate as well as buffering salts to the reaction medium gave almost 100% yield, leading to a simpler and more economical process. This was the first commercial example of an enzyme-catalysed reaction being used to produce a commodity chemical.

### (c) Whole-cell biocatalysis

Biocatalysis can also be performed using whole microorganisms. Three such commercial routes to vitamin B_2_, riboflavin, use *Ashbya gossypii*, a filamentous fungus (BASF), *Candida famata*, a yeast (ADM USA), or *Bacillus subtilis*, a Gram-positive bacterium (Roche) [119]. The earlier synthetic chemistry route required multiple steps, several solvent replacements and gave a maximum yield of 60%. The biocatalytic methods use less energy, reduce waste and use renewable resources, such as sugar or plant oil, as the starting materials and produce the riboflavin at approximately half the cost of the synthetic chemistry route.

## 6. Solvent selection for sustainability

When discussed in the context of the environment, solvents are usually seen as a problem to be overcome. However, it is possible for the selection of an appropriate solvent to provide a sustainable solution to a process problem. In the following sections, I attempt to show examples of how solvents have been used to deliver sustainable chemicals processes. These have been grouped by the advantage that the particular solvent provides.

## 7. The solvent is one of the reacting species

### (a) Asahi Kasei's polycarbonate synthesis

**Scheme 5.**
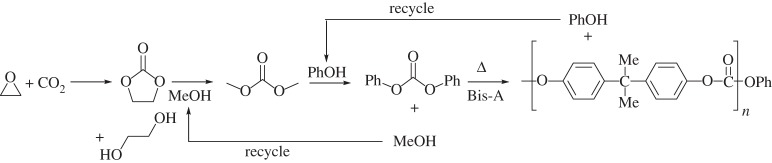
Polycarbonate synthesis.

The polymer most often referred to simply as polycarbonate (PC) is an aromatic carbonate polymer based on the monomer bisphenol-A (Bis-A). It has increased in use and importance with the spread of modern electronic devices. Asahi Kasei introduced a new process for the production of PC (scheme 5) that is acclaimed for replacing phosgene (COCl_2_) as the source of the carbonate link in the polymer with CO_2_ [120–122]. However, this process also led to the removal of dichloromethane (DCM) as a solvent. The new process is conducted in a ‘melt’ of the reaction mixture. While one might not choose one of the components to be the solvent for the others, this is undoubtedly a solution process.

In the original production of PC Bis-A dissolved in water reacts with phosgene dissolved in DCM. The reaction occurs at the interface of these two immiscible solutions. The DCM is a solvent for the PC product, thus maintaining a homogeneous solution throughout the process. However, the DCM is used in very large amounts (10× the amount of PC by mass). A similar mass of contaminated waste water is produced in this process (or 100× for optical grade PC). DCM also contaminated the product, leading to the release of this toxic solvent to the environment and a lower quality product. Also, although forming two layers, DCM has some solubility in water and water has some solubility in DCM, leading to energy-intensive and expensive separations.

The new process is conceptually simple. The overall reaction consists of ethylene oxide, CO_2_ and Bis-A to give PC and ethylene glycol. However, a number of intermediates are used to achieve this, which are either consumed or recycled in the process. First, the ethylene oxide and CO_2_ are reacted to give ethylene carbonate, which is then reacted with methanol to produce ethylene glycol (co-product) and dimethyl carbonate. The dimethyl carbonate is reacted with phenol to yield diphenyl carbonate and regenerate the methanol. A final transesterification reaction between diphenyl carbonate and Bis-A yields the PC and regenerates phenol [120].

Selling the ethylene glycol co-product of this reaction provides much of both the environmental and economic benefits of this process. With ethylene glycol being a co-product of the reaction, the atom economy of the reaction is 100% and the E-factor is 0 (assuming no waste from losses of recycled alcohols), compared with 80% and 0.24 if it had been a waste by-product. The new process saves energy and the capital cost of the plant for this process is less than half that of similar scale plants that use phosgene [120,122]. However, ethylene oxide is a hazardous material and phenol is environmentally harmful and the environmental impact of this synthesis is reliant upon containment of these.

### (b) PETRONAS's removal of mercury from natural gas

**Scheme 6.**
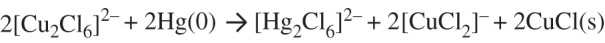
Oxidation of Hg by a chlorocuprate(II) ionic liquid.

An example of the use of the reactivity of a solvent to enable a process is the use of an ionic liquid to remove mercury from natural gas [123,124]. Fossil fuel production and use is a major source of environmental Hg pollution [125]. Mercury's corrosive nature can also lead to disastrous production plant failures [126]. The Hg is present in tiny concentrations in the gas stream, but the enormous volume of natural gas production leads to large absolute amounts of Hg passing into the production plant. Consequently, a Hg removal process that can operate at these low concentrations is required. This was solved by developing a chlorocuprate(II) ionic liquid system that was capable of absorbing the mercury and combining this with the supported ionic liquid phase (SILP) technology [123,127,128]. SILP technology was originally developed to enable catalysts dissolved in ionic liquids to be contacted with gaseous reactants [127]. The same ability enabled this Hg removal system to be brought to the full production plant scale. Full elucidation of the chemistry involved has proved difficult, but the inventors have deduced that scheme 6 is the most likely.

Some might question whether the production of natural gas can ever be thought of as sustainable. However, given that the use of natural gas is unlikely to significantly decline in the near future, it is vital that its production is conducted as sustainably as possible. The introduction of this technology has led (i) to a reduction in the pollution generated and (ii) to savings in the costs of the production of natural gas. This identifies it as a likely sustainable process (only time will tell). This is in spite of the fact that the ionic liquid itself would not be considered a ‘green’ solvent when considered in isolation from what it has enabled to be achieved.

### (c) BASF's nucleophilic HCl

**Scheme 7.**
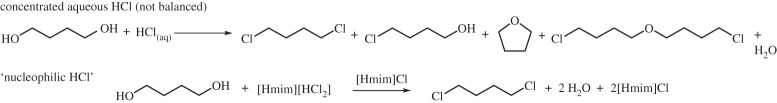
The chlorination of 1,4-butanediol.

The chlorination of alcohols requires reactants that do not produce water as a by-product, such as COCl_2_, SOCl_2_ or PCl_3_, etc. This is because the water produced as a by-product of the reaction forces the equilibrium back towards the starting alcohol. When the starting material is a diol, a number of possible partially chlorinated and ether by-products are formed. However, these are toxic, difficult to handle and environmentally damaging. BASF has recently commercialized an ionic liquid process for nucleophilic substitutions for the conversion of alcohols to halogenoalkanes that allows HCl to be used as the chlorinating agent [129]. When used in the chlorination of 1,4-butanediol this yields the dichloride without the formation of by-products (scheme 7).

In the nucleophilic HCl process, HCl is dissolved in a chloride ionic liquid, forming an [HCl_2_]^−^ salt [130]. This salt is the chlorinating agent. However, this does not explain why the water produced in the reaction no longer causes a problem. Spectroscopic investigations of water in ionic liquids show that it can interact very strongly with the ionic liquid's ions, particularly when the anion of the ionic liquid is a strong hydrogen bond acceptor, as is Cl^−^ [131–134]. These interactions lead to ionic liquids being able to stabilize water-sensitive solutes [135] or prevent water from reacting with a solute [136]. This behaviour is only possible when the ionic liquid is dry and the water level must be below 25 mol% for the reaction to be successful. The introduction of this process has led to the elimination of the highly toxic gas COCl_2_, with the attendant savings that derive from not needing to put in place the necessary engineering controls to handle it safely.

## 8. The solvent leads to a higher quality product

In the latter half of the twentieth century, health concerns over the effects of caffeine led to increased demand for decaffeinated coffee. Early forms of decaffeinated coffee were produced by caffeine extraction with dichloromethane [137]. The direct decaffeination of green coffee beans occurs before their roasting, which removed the DCM from the beans to levels of a few ppm. It was not the environmental concern that led to the replacement of this process. Alongside caffeine the DCM also removed important flavour components of the coffee, giving a poor quality product. This led to a number of other less environmentally concerning solvents being used for coffee decaffeination, but with the commercial driver being the search for a better product.

Ethyl acetate is an environmentally preferred solvent [80] used for coffee bean decaffeination [138]. First, the unroasted green beans are wetted with steam to increase their water content and to release the caffeine. Then the EtOAc is added to separate the caffeine from the moistened beans, from which residual EtOAc is removed by further steam treatment [137]. EtOAc is also used to decaffeinate tea [139].

Water has also been used to commercially decaffeinate coffee in the Swiss Water^®^ process [137]. The green beans are treated with hot water, which not only removes the caffeine, but also several other flavour chemicals. The caffeine is then extracted from the water with an activated charcoal filter. The water, still bearing many of the flavour chemicals, is reused for subsequent extractions of fresh beans. As this process is repeated, the water solution becomes saturated in the flavour compounds, so caffeine is extracted from the fresh beans, but the flavour compounds are not [140], giving a high-quality product. Many purveyors of water-decaffeinated coffee describe it as solvent-free processing and particularly point out the absence of EtOAc (a naturally occurring compound found in many fruits), targeting public misconceptions of ‘chemicals are bad for you’.

Supercritical CO_2_ (sc-CO_2_) decaffeination is also often described as solvent-free [137,141,142]. The green coffee beans are wetted and then the sc-CO_2_ is used to extract the caffeine. The sc-CO_2_ process is much more selective for the removal of caffeine than any of the other processes, leading to a high-quality product without the need for the additional steps to isolate it that are required for other methods. The start-up costs for an sc-CO_2_ decaffeination plant are higher than those of the other methods, but the economic viability of the sc-CO_2_ process is enhanced because the caffeine is a saleable co-product, particularly as it can be labelled as ‘natural’, for use in products such as cosmetics and so-called ‘energy’ drinks for which this label can carry a premium [143]. Sc-CO_2_ processing has become a widely used method in the food industry, such as in the decaffeination of tea [139], the removal of fat to produce low-fat varieties, the removal of alcohol to produce low-alcohol beers and wines and the removal of pesticides from rice and the extraction of flavours and fragrance compounds [144,145].

## 9. The solvent selection enables a reduction in the number of synthesis steps

The number of intermediate product isolations in a multi-step chemical synthesis can greatly negatively affect the environmental impact of a process. This usually occurs because individual steps are independently optimized and then connected in a chain of reactions to yield the final product. Thus, one step can be followed by another with the solvent for the first being unsuitable for the second. However, it may be possible to select a solvent so that it is capable of supporting several consecutive reactions and lead to a significant reduction in the waste generated by the overall process.

### (a) Pfizer's sertraline synthesis

**Scheme 8.**
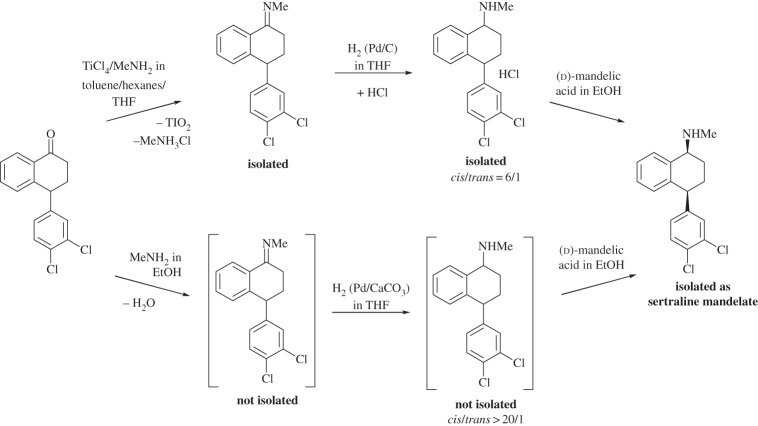
The synthesis of sertraline [146].

The use of ethanol, together with adjustment of the synthetic route, allowed the final three steps of Pfizer's sertraline synthesis (scheme 8) to be conducted without intermediate product isolation [146,147]. The first commercial route used 101.4 l of solvent for every kilogram of product isolated (34 l EtOH, 28.4 l, EtOAc, 19 l THF, 8 l toluene and 12 l hexane). Most of these were used in the purifications of the isolated intermediates.

The new synthesis changed the reactions to affect each transformation rather than change the intermediates in the process. It avoided the use of TiCl_4_ and eliminated TiO_2_ waste, removing the need for a costly and wasteful filtration. Ethanol was not the optimum solvent choice for this step if considered in isolation, showing the importance of considering the overall process, not just the individual parts. The improved reduction of the imine intermediate to give a *cis*/*trans* product ratio of 20:1 instead of 6:1 in the old procedure gave an inherently more efficient reaction and avoided the need for repeated recrystallizations, so that the final enantiomerically pure sertraline mandelate could be isolated, this resolution now being the most wasteful step. Altogether this led to a reduction in solvent use to 24 l kg^−1^ product (15 l EtOH and 9 l EtOAc).

It is not possible from the available information [146,148] to compare the details of the performances achieved in these processes in order to calculate their green metrics, but it is possible to estimate the low end of the likely range of values. The original process used 84 kg kg^−1^ product of combined solvents and generated 4.4 kg kg^−1^ of TiO_2_–MeNH_2_⋅HCl and 0.4 kg kg^−1^ of the *trans*-imine, equating to a PMI of ≈90. In the new method, the only by-product of the imine formation is water, so the only waste of any significance comes from the solvents used, giving a PMI of ≈21. These calculations assume that there is no solvent recovery in either process. It has been estimated that the reduction of waste for this new route saves Pfizer over $100 000 pa [88,149].

### (b) Merck's sitagliptin synthesis

The first-generation synthesis of sitagliptin, a treatment for type 2 diabetes, was conducted in multiple steps [150]. First 3-trifluoromethyl-[1,2,4]triazolo[4,3-a]piperazine was prepared, so that it could be reacted with the hydrolysed form of the lactam *N*-benzyloxy-4(*R*)-[1-methyl-(2,4,5-trifluorophenyl)]-2-oxoazetidine. It is the formation of this lactam intermediate and its subsequent reaction with the triazole that was redesigned for the second-generation synthesis.

**Scheme 9.**
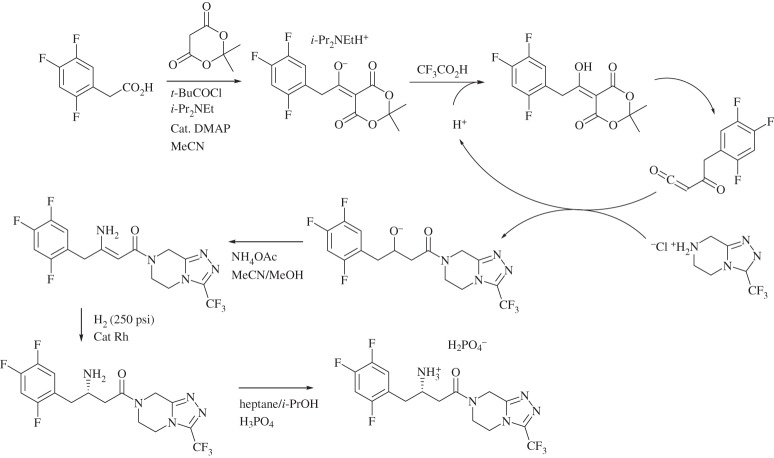
One-pot synthesis of sitagliptin [151].

The original synthesis required three isolations (including the product), two aqueous–organic liquid separations and two solvent switches. This synthesis was replaced with multi-step one-pot synthesis in high concentration in acetonitrile (scheme 9) [151]. This process led to a reduction in the E-factor from 250 to 50 for the overall synthesis, including a complete elimination of organic-contaminated aqueous wastes.

**Scheme 10.**
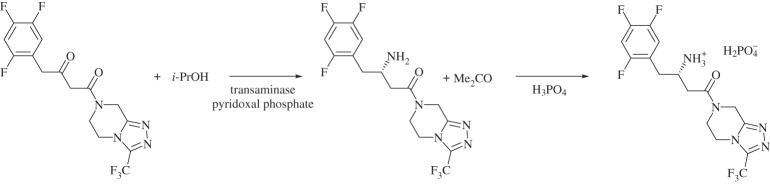
Enzymatic synthesis of sitagliptin [152].

Despite this process being a considerable improvement over its predecessor, the late-stage hydrogenation was only moderately stereoselective and required high-pressure conditions [151]. The removal of the metal catalyst by absorption onto a polymer impregnated with activated carbon and the final recrystallization as the [H_2_PO_4_]^−^ salt led to reduced yield [151]. The final version of the sitagliptin synthesis avoided this hydrogenation by using a transaminase enzyme to directly aminate the prositagliptin diketone precursor with *iso*-propylamine (scheme 10) [152], giving a highly enantiopure product. The enzymatic process gives a 10–13% increase in overall yield, a 53% increase in productivity (kg l^−1^ day^−1^), a 19% reduction in total waste and the elimination of all heavy metals. In addition to these environmental advantages, the biocatalytic process eliminated the need for specialized high-pressure equipment, leading to reductions in both capital and running costs.

## 10. The solvent leads to a reduction of by-product formation

### (a) Novartis's synthesis of 4-cyano-1,2,3-triazoles

**Scheme 11.**
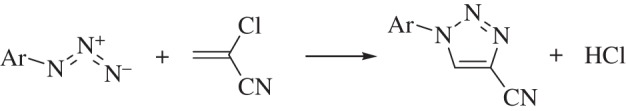
The formation of 4-cyano-1,2,3-triazoles.

The concept now known as ‘Click’ chemistry has had a growing importance during the early years of this century [153]. It uses readily available reactive starting materials in reliable reactions to give high yields. The cycloaddition reactions of azides have been particularly of interest. However, these reactions are not always straightforward. One such case is the formation of 4-cyano-1,2,3-triazoles from organic azides and 2-chloroacrylonitrile (scheme 11) [28]. The initial 1,3-dipolar cycloaddition yields a triazoline which eliminates HCl to form the triazole product. If conducted in a single homogeneous solution, yields are disappointing, because the by-product HCl reacts with 2-chloroacrylonitrile to initiate its polymerization. Controls such as conducting the reaction at low concentration of starting materials and with excess 2-chloroacrylonitirle are not effective and lead to polymeric waste. Conducting the reaction in the presence of water improves the yield to 98% [154]. This is because the starting materials and product are not soluble in water, but HCl is. As the HCl is generated it is rapidly dissolved in the water, removing it from the reaction solution so that it cannot initiate the 2-chloroacrylonitrile polymerization. This kind of reaction is now often described as ‘on-water’ [155].

### (b) ICOS Corporation/Eli Lilly's synthesis of tadalafil

The synthesis of tadalafil, a treatment for erectile dysfunction, begins with a Pictet–Spengler reaction of tryptophan methyl ester. The medicinal chemistry route started with (±)-tryptophan methyl ester, used DCM as the solvent and yielded the cyclic product in both *cis*- and *trans*- forms, which were separated by flash chromatography with the desired *cis*-isomer having a maximum yield of 42% [156]. By replacing the DCM with *iso*-propylalcohol and starting with d-tryptophan methyl ester the *cis*-isomer could be obtained in high yield. Both isomers are formed during the reaction, but the *cis*-isomer is poorly soluble in the *i*-PrOH and spontaneously precipitates, leaving the *trans*-isomer in solution. However, the two isomers are in equilibrium in solution, so heating the solution over time generates more of the *cis*-isomer, which precipitates further and so on until the reaction is complete. This elimination of by-product formation led to a dramatic reduction in the waste formed and eliminated the need for flash chromatography, hence greatly reducing solvent use.

## 11. The solvent enables product separation

### (a) BASF's BASIL (biphasic acid scavenging utilizing ionic liquids) process

**Scheme 12.**
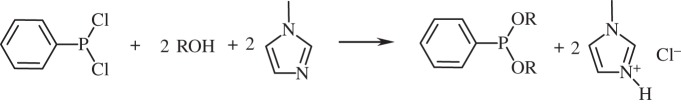
The BASIL synthesis of alkoxyphenylphosphanes.

BASF produces alkoxyphenylphosphanes as the raw materials for a range of UV-photoinitiators. Originally, Et_3_N was used as a proton scavenger, leading to the formation of [Et_3_NH]Cl. The alkoxyphenylphosphanes are liquid and the [Et_3_NH]Cl solid, resulting in a thick slurry that required separation using filter presses that regularly blocked. The BASIL process (scheme 12) solved this by replacing the Et_3_N with 1-methylimidazole, which gives 1-methylimidazolium chloride ([HC_1_im]Cl, mp=75°C) with the HCl formed, which separates spontaneously as a second liquid phase under the reaction conditions [157,158]. This eliminated the costly and unreliable filtration step. The by-product [HC_1_im]Cl is deprotonated to recycle the 1-methylimidazole, again reducing costs.

1-Methylimidazole is also a nucleophilic catalyst [159]. This enabled the development of a new jet stream design for the new all-liquid BASIL^^TM^^ reactor, which gave an increased productivity of a factor of 8×10^4^ to 690.000 kg m^−3^ h^−1^, giving significant cost savings. A recent ecoefficiency analysis has shown that the BASIL technology is far more environmentally sustainable than the process using tertiary amines (http://www.BASFSE.com/group/corporate/en/function/conversions:/publish/content/sustainability/eco-efficiency-analysis/images/BASFSE_Eco-Eff-iciency_Label_Basil_2005.pdf).

## 12. Catalysts separation and recycling

Homogeneous catalysis is inherently more efficient (all metal centres are involved in catalysis, flexibility of ligand design to optimize catalyst performance, etc.) than using solid catalysts. Despite this, solid catalysts are usually preferred. This is because it can be very difficult and costly to separate a homogeneous catalyst from the reaction products. One approach to solving this problem is biphasic catalysis [160–163].

In aqueous/organic biphasic systems, the reactants and products are soluble in the organic phase but largely insoluble in the aqueous phase, while the catalyst is insoluble in the organic phase but soluble in the aqueous phase. Thus, the separation of the catalyst from the reaction products is achieved. The reactants are contacted with the catalyst by rapid stirring to give a useful rate, with the reaction occurring at the liquid–liquid interface, not by transfer into one or the other bulk phases [164].

### (a) Ruhrchemie–Rhône-Poulenc's hydroformylation process

**Scheme 13.**
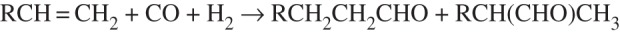
Olefin hydroformylation.

The most successful aqueous/organic biphasic catalysis process is the Ruhrchemie–Rhône-Poulenc lower olefin hydroformylation (scheme 13) [165]. This uses a water-soluble form of Wilkinson's homogeneous hydroformylation catalyst, with a sulfonated triphenylphosphine ligand, [RhH(CO){(*m*-SO_3_NaC_6_H_4_)_3_P}_3_], initially for the hydroformylation of propene to butanal [166]. This process replaced a previous industrial process, which used a cobalt catalyst at high pressure, giving several advantages including: excellent selectivity to linear aldehydes, simpler process operation, efficient catalyst recycling and reduced energy demand. As well as giving an improved commercial performance this reduced the environmental impact with the biphasic process having an estimated E-factor of 0.04–0.1, compared with 0.6–0.9 for the high-pressure cobalt process [160,163].

### (b) Asahi Kasei's hydrogenation of benzene to cyclohexene

Aqueous biphasic conditions can also be used with heterogeneous catalysts. Asahi Kasei has commercialized a process for the hydrogenation of benzene to cyclohexene [167]. The hydrogenation takes place in an aqueous phase that is in contact with a solid ruthenium catalyst. While benzene forms a separate phase from the water, it is sufficiently soluble in water to be contacted with the catalyst. The less soluble cyclohexene product transfers to the benzene phase before it can react further, preventing the formation of cyclohexane.

### (c) The Shell higher olefin process

The Shell higher olefin process (SHOP) uses an organic/organic biphasic system to separate its catalyst from its products [168,169]. In SHOP, ethene is oligomerized to α-olefins using a nickel catalyst. 1,4-Butanediol is a good solvent for both the catalyst and the ethene starting material, but a poor solvent for the product mixture, which separates as a second liquid phase. Key to both the environmental and commercial success of this process is the ability to separate the immediately saleable C_11_−C_14_ α-olefins from the non-saleable portions of lighter and heavier olefins, which can then undergo isomerization and metathesis [170] reactions to generate a new set of C_11_−C_14_ monoolefins for sale. The process can be tuned to produce any preferred product distribution.

SHOP replaced earlier thermal cracking of petroleum-derived wax. SHOP is much more selective to the desired linear C_11_−C_14_ α-olefins and hence less wasteful. SHOP was introduced to meet increased demand for linear α-olefins of this range to solve an environmental pollution problem. These α-olefins are precursors to surfactants used as both domestic and industrial detergents. These had formerly been branched-chain ‘hard’ detergents, which could not be biodegraded, causing significant pollution problems. The replacement of these with biodegradable linear surfactants created the demand for large amounts of linear α-olefins and hence the need for a new process [168].

## 13. Being green is not enough

Having a low environmental impact is necessary for a product or process to be sustainable, but it is not on its own sufficient for it to be so; it must also be a commercial success. There are a number of examples of technically excellent processes that have been introduced, only later to be withdrawn due to commercial pressures.

One such example is Thomas Swan Ltd's hydrogenation of isophorone in sc-CO_2_ over a supported palladium catalyst [171]. The sc-CO_2_ system gave selective hydrogenation of isophorone to 3,3,5-trimethylcyclohexanone, with no 3,3,5-trimethylcyclohexanol or 3,3,5-trimethylcyclohexane by-products [172,173]. This eliminated an expensive and energy-intensive separation of these from the product. From 2002 to 2009, Thomas Swan & Co. ran a commercial production plant operating at a 100 kg h^−1^ scale, after which demand for the product fell and the plant was taken out of production [171]. A similar fate befell the Eastman Chemical Company process for the isomerization of 3,4-epoxybut-1-ene to 2,5-dihydrofuran in a phosphonium iodide ionic liquid [44,47].

The cost of the implementation of a new technology can also prevent a technically excellent process from being adopted because of commercial pressures. One of the earliest potential large-scale applications of ionic liquids was the Institut Français du Pétrole Difasol process [174,175]. This is a biphasic process for the dimerization of olefins, in which a nickel catalyst is dissolved in an ionic liquid phase with the ionic liquid acting as both solvent and co-catalyst. The product is separated as a liquid layer that forms above the ionic liquid. The Difasol process can either be used as an addition to the previous homogeneous Dimersol process or as a replacement for it. Despite the fact that the Difasol process offers more efficient catalyst use, higher yield, better dimer selectivity, enhanced reactor space time yield and energy savings over its predecessor, it appears that the cost of capital equipment has prevented it from yet being put into commercial application.

## 14. Conclusion

The environmental concerns that surround the use of solvents for chemicals processing will ensure that this remains an active area for research for some time to come. The examples that I have shown above demonstrate that it is possible to make considerable advances in the reduction of the amounts of solvents used in chemicals processing. They also go beyond this to demonstrate the potential of appropriate solvent selection to improve other areas of a process's performance and hence its overall sustainability. These examples also demonstrate that the *implementation of the concept of sustainability in the production and use of chemicals and chemical products* requires that chemicals processing must be both environmentally and commercially sustainable. Furthermore, reducing the cost of chemicals production and hence the price of chemicals is vital for *the application of chemistry and chemical products to enable sustainable development*. The successful introduction of a truly sustainable chemicals industry is one of the great challenges that we face today. There are relatively few examples described in the open literature of the introduction of processes based upon sustainable solvent use, particularly when compared with the thousands of commercial chemical processes that exist. This does not necessarily mean that so few have been implemented; it is likely that some companies have chosen to hide these behind a wall of commercial confidentiality. It would, however, be helpful to see more of these described so that they can act as an inspiration to others trying to achieve this important aim for us all.
